# Long Term Survival of Heritable Pulmonary Arterial Hypertension Associated with Hereditary Hemorrhagic Telangiectasia: A Case Series

**DOI:** 10.3390/jcm13010141

**Published:** 2023-12-27

**Authors:** Parth Jamindar, Michael Pope, James Gossage

**Affiliations:** 1 Division of Pulmonary, Critical Care and Sleep Medicine, Augusta University Medical Center, Augusta, GA 30912, USA; parth.jamindar@gmail.com (P.J.); jgossage@augusta.edu (J.G.); 2 Division of Internal Medicine, Augusta University Medical Center, Augusta, GA 30912, USA

**Keywords:** hereditary hemorrhagic telangiectasia, pulmonary hypertension, pulmonary arterial hypertension, right heart catheterization

## Abstract

Hereditary hemorrhagic telangiectasia (HHT) is a hereditary disease characterized by recurrent epistaxis, mucocutaneous telangiectasias, and visceral arteriovenous malformations. Multiple genetic mutations have been linked to this rare disease, including ENG, ALK1 (ACVRL1), and MADH4. Pulmonary hypertension is a potential complication of HHT, with the most common phenotypes being World Health Organization (WHO) group 1 heritable pulmonary arterial hypertension (PAH), which is typically associated with ALK1 mutation; WHO group 2 pulmonary hypertension due to high output heart failure from hepatic arteriovenous malformations and/or anemia; and WHO group 2 due to high pulmonary artery wedge pressure. There is scarce evidence to help guide treatment of heritable PAH in HHT, and observational literature suggests that patients with HHT and heritable PAH have a worse prognosis compared to patients with idiopathic PAH. We describe the diagnosis, pulmonary hemodynamics, and detailed treatment courses of three patients with ALK1-associated HHT and PAH, who all exhibited objective clinical improvement with parenteral prostacyclins and oral agents.

## 1. Introduction

Hereditary hemorrhagic telangiectasia (HHT) is a hereditary disease characterized by recurrent epistaxis, mucocutaneous telangiectasias, and visceral arteriovenous malformations (AVMs) [1,2]. This rare disease is known to be caused by genetic mutations in ENG, ALK1 (ACVRL1), MADH4, and perhaps others [3]. Pulmonary hypertension (PH) is a potential complication of HHT, with a frequency of 1.5–31% depending on the study population and the method of diagnosis [4,5,6,7]. The most common phenotypes of PH in HHT include WHO group 1 heritable pulmonary arterial hypertension (PAH), which is typically associated with an ALK1 genetic mutation; WHO group 2 PH due to high output heart failure from hepatic AVMs and/or anemia; and WHO group 2 PH due to high pulmonary artery wedge pressure (PAWP) [4,8]. Based on current observational literature, patients with PAH associated with HHT (HHT-PAH) have a worse prognosis compared to idiopathic PAH even though the former seem to have similar presenting hemodynamics on right heart catheterization (RHC) [9].

We describe the diagnosis and detailed treatment courses of three patients with definite HHT associated with ALK1 mutations who developed PAH. With aggressive treatment of their PAH using a combination of parenteral prostacyclins and oral agents, these patients had significant improvement in PH symptoms, pulmonary hemodynamics, and 6 min walk distance (6MWD), as well as long-term survival on follow-up.

## 2. Methods

The goal of this study was to review the outcome of treatment for PAH in patients with definite HHT treated at our center. We reviewed the records of all patients who had suspected HHT and who underwent RHC at Augusta University Medical Center (AUMC) between 1 January 2005 and 30 August 2023. HHT was generally suspected based on some combination of recurrent spontaneous nosebleeds, mucocutaneous telangiectasias, visceral AVMs, and positive family history of HHT. RHC was typically performed due to the presence of PH by echocardiogram (sPAP estimated at ≥40 mmHg) and/or symptoms and signs of PH. Patients were characterized as having definite HHT according to the Curaçao criteria [1]. PAH was diagnosed based on RHC showing a mean pulmonary artery pressure (mPAP) > 20 mmHg, a PAWP ≤ 15 mmHg, and a pulmonary vascular resistance (PVR) > 2 Wood units (WU), as well as the absence of other causes for pulmonary hypertension such as left heart disease or COPD [10].

## 3. Results

**Figure 1:**
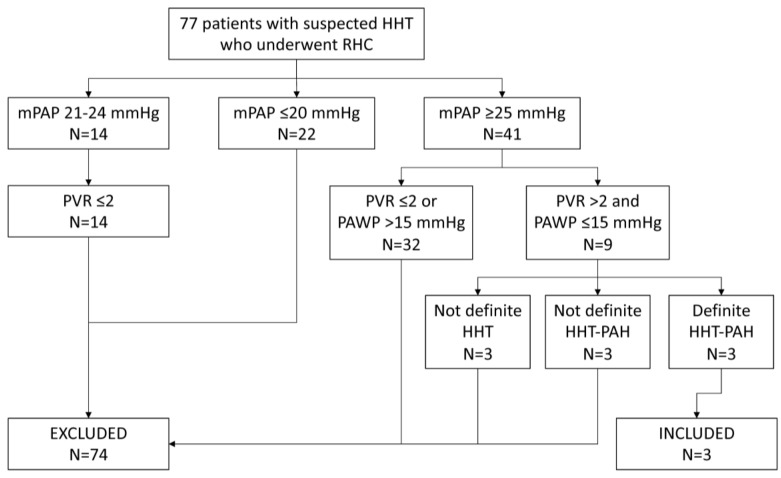
Subject selection route.

We identified 77 unique patients who had suspected HHT and who underwent RHC (Figure 1). Fourteen patients had an mPAP of 21–24 mmHg; all 14 had a PVR ≤ 2 WU and 6 had a PAWP ≤ 15 mmHg. Forty-one patients had an mPAP of ≥25 mmHg, nine of whom had a PVR > 2 WU and a PAWP ≤ 15 mmHg. Three of these latter patients had definite HHT and hemodynamics that were consistent with PAH (mPAP 49 mmHg and PVR 4.1 WU, mPAP 32 mmHg and PVR 3.6 WU, and mPAP 36 mmHg and PVR 3.7 WU) but had factors such as COPD or high positive ANA that confounded the diagnosis of PAH related to HHT and thus were excluded; these patients were lost to follow-up. Three patients had clearcut PAH but did not have definite HHT (2 had negative genetic testing for the 3 typical mutations) and were excluded.

We present the case vignettes for the remaining three patients with definite HHT and PAH, all of whom were diagnosed as having HHT-PAH due to a pathogenic ALK1 mutation. All three patients had negative HIV and connective tissue disease screening, normal ventilation/perfusion lung scans, and unremarkable pulmonary function testing. No patient had a cardiac index (CI) of >3.5 L/min/m^2^ at baseline. At the time of this writing, two patients had survived 180 and 247 months after initial diagnosis and one had died 45 months after initial diagnosis.

### 3.1. Patient 1

A 38-year-old female was referred to the AUMC HHT Center for evaluation of PH. She was previously diagnosed with PH but had not yet had an RHC or a formal evaluation for HHT. On presentation to the AUMC, she complained of chronic epistaxis and progressive WHO functional class II–III exercise intolerance. The family history of HHT was significant in her father and cousin. A physical exam showed significance for multiple mucocutaneous telangiectasias, a jugular venous pressure (JVP) of 8 cm, and trace leg edema. A CT scan demonstrated three small pulmonary AVMs and multiple hepatic AVMs with a proper hepatic artery diameter of 6.7 mm. Her 6MWD was 540 m. The RHC at that time (Table 1) showed a right atrial pressure (RAP) of 12 mmHg, an mPAP of 53 mmHg, a PAWP of 7 mmHg, and a PVR of 10.2 WU. She had a left-to-right shunt of 1.38 L/min and a Qp/Qs of 1.42 due to hepatic AVM, but this was not thought to be the main cause of her PH. There was no significant vasodilator response to inhaled nitric oxide of 40 ppm. She was started on 20 mg sildenafil t.i.d.

Three months after initial referral, she was hospitalized for right heart failure (RHF) and started on bosentan and IV epoprostenol, which was titrated in quantities of up to 13 ng/kg/min over nine days. A repeat RHC confirmed a severely elevated RAP (Table 1), which was thought to be partly due to increased cardiac output from rapid uptitration in epoprostenol. Notably, cardiac output had increased from 4.5 L/min at baseline to 6.6 L/min. She continued with aggressive diuresis along with downtitration of epoprostenol to 9 ng/kg/min and was discharged with symptomatic improvement. A repeat outpatient RHC 6 months later showed significant improvement in all measures (Table 1). Three months later, she was transitioned from epoprostenol to inhaled treprostinil in nine breaths q.i.d. at her request and from bosentan to ambrisentan due to elevated liver enzymes. One hundred sixty-four months after her initial diagnosis, she was WHO functional class I with a 6MWD of 795 m, a B-type natriuretic peptide (BNP) of 36 pg/mL, and an sPAP of 28 by echocardiogram. One hundred eighty months after her initial diagnosis, she was WHO functional class I.

### 3.2. Patient 2

A 40-year-old female was referred to the AUMC for continued management in a PAH research study. She was diagnosed with PAH at an outside hospital by RHC four years prior, which showed a RAP of 13 mmHg, an mPAP of 56 mmHg, a PAWP of 15 mmHg, and a PVR of 8.7 WU. She was started on bosentan at the time of her initial diagnosis and continued this therapy for 14 months. She then started sitaxentan as part of a research trial. Subcutaneous treprostinil was started five months later, and then she transitioned to IV treprostinil after one year, which was slowly titrated up. Sildenafil was later added.

At the time of referral to the AUMC, her medications included sitaxentan, sildenafil, and IV treprostinil of 60 ng/kg/min. Evaluation was significant for frequent epistaxis and mucocutaneous telangiectasias, and there was a family history of HHT in her father. Therefore, a diagnosis of HHT-PAH was determined and later confirmed with genetic testing showing an ALK1 mutation. She was WHO functional class II with a 6MWD of 552 m. Sitaxentan was stopped shortly after her referral due to early closure of the research study by the sponsor. A repeat RHC 53 months after initial diagnosis showed continued unfavorable hemodynamics (Table 1) with no significant vasodilator response to inhaled nitric oxide of 40 ppm; therefore, the patient was started on ambrisentan. She had no noticeable improvement in functional status or 6MWD on this regimen. Ambrisentan was switched to bosentan after seven months, but she again showed no improvement, so the bosentan was discontinued five months later. Serial echocardiograms over the next two years demonstrated persistent severe right ventricular (RV) enlargement and elevated right ventricular systolic pressures (RVSP) of 118 mmHg. A repeat RHC 86 months after initial diagnosis demonstrated unchanged hemodynamics (Table 1), with a left-to-right shunt of 2.16 L/min and a Qp/Qs of 1.42 due to hepatic AVM.

With gradual uptitration of treprostinil, the patient demonstrated improvement in symptoms and functional status. Sildenafil was switched to tadalafil due to insurance issues. A repeat RHC 114 months after initial diagnosis showed mildly improved but still suboptimal hemodynamics (Table 1), with a left-to-right shunt of 1.73 L/min and Qp/Qs of 1.41. The treprostinil dose was again uptitrated and the WHO functional class remained at II.

Approximately 226 months after initial diagnosis, she noted increased dyspnea and BNP was elevated to 129 pg/mL. A repeat RHC 1 month later showed worse hemodynamics (Table 1), though the BNP and left-to-right shunt (0.8 L/min) were improved. Over the next 18 months, she demonstrated a stuttering course, with overall worsening in symptoms, 6MWD, and BNP despite judicious titration of diuretics and treprostinil. She was referred for lung transplant evaluation, which found evidence of portal hypertension, including upper abdominal collaterals on an abdominal MRI and a portal gradient of 15 mmHg. Portal hypertension was attributed to regenerative hyperplasia, which can be seen in HHT. In an effort to treat the portal hypertension and improve her candidacy for a lung transplant, she was started on six doses of 5mg/kg of intravenous bevacizumab (236 months after initial diagnosis) every 2 weeks, followed by one dose every 6 months. Her most recent RHC 245 months after initial diagnosis showed mildly worse hemodynamics (Table 1); at that time, she was functional class III with a 6MWD of 391 m and a BNP of 243 pg/mL. Diuretics were increased, with some improvement. Two hundred forty-seven months after her initial diagnosis, she was WHO functional class III.

### 3.3. Patient 3

A 38-year-old female was referred to the HHT Center for management of HHT. She was diagnosed with PAH at age 37 after developing progressive dyspnea that required hospitalization. RHC during that admission confirmed the diagnosis of PAH (RAP 17 mmHg, mPAP 69 mmHg, PAWP 13 mmHg, and PVR 8.4 WU). She was started on ambrisentan and tadalafil. She then discontinued tadalafil due to worsening epistaxis and started selexipag. She underwent a repeat RHC at a different hospital 10 months after her initial diagnosis, which showed minimally improved hemodynamics (Table 1) on ambrisentan and selexipag. Her regimen was then switched to ambrisentan, riociguat, and 9 puffs q.i.d. of inhaled treprostinil before presenting to our clinic.

At the time of referral to the AUMC, her HHT manifested as epistaxis, mucocutaneous telangiectasias, hepatic AVMs, and iron deficiency anemia. She was WHO functional class II with a 6MWD of 447 m. Her inhaled treprostinil was uptitrated to 12 puffs q.i.d. She underwent a repeat RHC 18 months after her initial diagnosis, which demonstrated improved pulmonary hemodynamics (Table 1) with no left-to-right shunt. Thirty-six months after her initial diagnosis, she was WHO functional class II with a 6MWD of 459 m and a BNP of 90 pg/mL.

Forty to forty-one months after initial diagnosis, she noticed increasing weight, dyspnea, and epistaxis, and Hgb decreased to 6.7 g/dL. She was treated with intravenous loop diuretics, red blood cell transfusion, and IV iron, with some improvement in symptoms; Hgb increased to 9.4 g/dL. Forty-two months after initial diagnosis, she was hospitalized at the AUMC for RHF and treated with loop diuretics and nasal sclerotherapy; a transition to SQ treprostinil was discussed, but she was not ready for that. During the next 4 months, she required increased diuretics for RHF; Hgb fell to 6.4 g/dL and she required additional red blood cell transfusions and IV iron. Outpatient EGD showed “a few non-bleeding gastric AVMs”, and oral tranexamic acid of 1300 mg t.id. was started. Forty-five months after initial diagnosis, she was admitted to a hospital in her hometown for RHF. She initially improved with increased diuretics. Our center recommended consideration of RHC followed by SQ treprostinil, but she was lost to follow-up and died 2 weeks later.

## 4. Discussion

Our study is the first series to carefully document the detailed clinical and hemodynamic response to treatment of ALK1-related heritable PAH. All three patients had definite HHT and clear-cut PAH with normal CI at baseline. All patients received aggressive treatment with a three-drug regimen including parenteral prostacyclins, although only two patients remained on the three drugs at the time of final follow-up. Finally, all patients showed improvement in PAH symptoms, 6MWD, mPAP, and PVR—improvements that were prolonged in two out of three. At the time of last follow-up, patient 1 remained functional class I. However, patient 2 showed signs of deterioration after approximately 19 years and was being evaluated for lung and liver transplant. Her picture was complicated by portal hypertension, which was thought to be due to regenerative hyperplasia rather than hepatic AVM with high-output heart failure. Whether she now has a component of portopulmonary hypertension is unclear. Patient 3 did well for approximately 3.5 years but then deteriorated fairly quickly with RHF, which was possibly exacerbated by anemia. Although she received inhaled treprostinil, a transition to an SQ or IV prostacyclin in the final months may have been beneficial.

Previous reports of HHT-PAH have suggested poor survival. Girerd et al. compared 32 patients with an ALK1 mutation to 93 with a BMPR2 mutation and 277 with idiopathic PAH and found that ALK1 patients had earlier presentation and shorter survival compared to the other groups [9]. Five of the nine ALK1 patients in the French PAH Network died from PAH after 1–73 months, whereas four were still alive after 9–81 months of treatment [9]. In a study of nine patients with HHT-related PAH, Li et al. reported a survival of 77.8% at one year and 53.3% at three years [11]. The longest reported survival of ALK1-related PAH that we could find was approximately 25 years in a patient treated with multiple agents, including subcutaneous prostacyclin and low-dose tacrolimus [12]. This patient, along with patients 1 and 2 from our series, represent the three longest survivals reported in the literature. Revuz et al. reported three patients with HHT-PAH and a survival of 120–191 months, but two of those patients had a cardiac index >4.0 L/min/m^2^ and the third was 8 years old and had a cardiac output of 4.69 L/min, which is suggestive of at least a component of group 2 PH in all three [4]. Our patients differed in some ways from those in the Girerd series. All of our patients were diagnosed with PAH at age 36–38 years, whereas the mean age of diagnosis in the Girerd series was age 27 [9]. Also, we included only patients with a clearly normal cardiac index, whereas several other series included some patients with cardiac indices >4.0 L/min/m^2^, which potentially could have included some patients who had mixed group 1 and 2 PH related to high-output heart failure from hepatic AVM [4,5,9].

There is scarce evidence to help guide management of heritable PAH in HHT [3]. Two case reports demonstrated symptomatic and hemodynamic improvement in three patients treated with bosentan [13,14]. Therapies initiated in the French PAH Network included monotherapy or combination therapy with parenteral prostacyclins, bosentan, and sildenafil, though no one received a three-drug combination [9]. Of those who survived, there were no follow-up RHC data [9]. Lyle et al. reported that eight of their HHT-PAH patients received PH-specific therapy, but only two received prostacyclins and there were few clinical follow-up data [5]. Li et al. reported nine patients with HHT-PAH but provided no details of treatment [11]. Although our series is too small to draw conclusions about optimal therapy, it does suggest that long-term survival is possible with aggressive combination therapy that includes parenteral prostacyclins.

Several papers have expressed concern for increased bleeding risk when HHT-PAH patients are treated with various PH drugs [5,12]. Lyle et al. noted increased epistaxis or GI bleeding in five patients during treatment for PAH [5]. Although all of our patients suffered from epistaxis, it was generally manageable with the usual measures, including oral doxycycline, endonasal cautery, and endonasal sclerotherapy. Although patient 3 had increasing problems with epistaxis in her final 6 months, it was unrelated to changes in PAH medication. In our opinion, concerns that PAH medications may increase epistaxis should not limit the use of these potentially life-saving medications.

Finally, it is important to fully characterize the hemodynamics in each patient with HHT-associated PH. As noted in the introduction, the most common phenotypes of PH in HHT include WHO group 1 heritable pulmonary arterial hypertension, WHO group 2 PH due to high-output heart failure from hepatic AVMs and/or anemia, and WHO group 2 PH due to high PAWP [4,8]. RHC is an essential early step to confirm PH and assess PAWP, CI, and PVR. If the PVR is normal, there is no role for PAH medications. When the CI is high (typically ≥4 L/min/m^2^) and hepatic AVMs are present, consideration should be given to treatment of hepatic AVMs with IV bevacizumab or liver transplantation, especially if RAP and/or PAWP are also elevated. In our database analysis, 28 of 55 patients with a mPAP > 20 mmHg had a PAWP > 15 mmHg and a normal PVR, and several of them were treated for hepatic AVM. If PH is mild and CI is <4 L/min/m^2^, treatment of hepatic AVM is often deferred. In some cases, an elevated CI may be due to anemia and may respond to correction of anemia with treatment of HHT-related bleeding and iron supplementation.

In conclusion, all patients in this series showed initial improvement in PAH symptoms, RHC parameters, and 6MWD, and two patients showed long-term survival. Aggressive medical management of HHT-PAH, especially the use of parenteral prostacyclins, may lead to increased survival and improved symptomatic outcomes.

## Figures and Tables

**Table 1:** Hemodynamic data *.

	PAH	RAP	mPAP	PAWP	PVR	CO **	BNP	6MWD	Hgb
	Treatment	(mmHg)	(mmHg)	(mmHg)	(WU)	(L/min)	(pg/mL)	(meters)	(g/dL)
Patient 1									
Baseline	0	12	53	7	10.2	4.5	521	540	11.6
3 months	B,S,E13	24	54	11	6.5	6.6	421	363	10.3
9 months	B,S,E13	3	25	5	2.9	6.9	4	660	11.8
Patient 2									
Baseline	0	13	56	15	8.7	4.7	-	-	-
53 months	S,T60	6	55	13	7.6	5.5	22	552	14.8
66 months	A,S,T60	5	50	10	7.1	5.6	6	551	15.4
86 months	S,T72	7	53	13	7.3	5.5	24	548	15.1
114 months	S,T87	7	48	12	5.6	6.4	30	561	15
227 months	S,T105	10	57	18	6.2	6.3	79	515	15.3
245 months	S,T115	16	58	13	6.1	7.3	243	391	14.7
Patient 3									
Baseline	0	17	69	13	8.4	6.7	-	428	13.9
10 months	A,Se	8	67	12	9.2	6	57	334	-
18 months	A,R,IT9	8	61	9	6.6	7.9	53	447	14.6

A = ambrisentan, B = bosentan, E = epoprostenol, Hgb = hemoglobin, IT = inhaled treprostinil, R = riociguat, S = sildenafil, Se = selexipag, T = IV treprostinil; E and T are followed by dose in ng/kg/min; IT is followed by puff/dose. * 6MWD and BNP were included if within 3 months of the date of RHC and if there had been no overt clinical changes. ** CO (cardiac output) measured by thermodilution method except at 245 months in patient 2, in whom it was measured by Fick.

## Data Availability

All data generated or analyzed during this study are included in this manuscript.

## Abbreviations

ANA	Antinuclear antibody
AUMC	Augusta University Medical Center
AVM	Arteriovenous malformation
BNP	B-type natriuretic peptide
CI	Cardiac index
CO	Cardiac output
COPD	Chronic obstructive pulmonary disease
EGD	Esophagogastroduodenoscopy
HHT	Hereditary hemorrhagic telangiectasia
HIV	Human immunodeficiency virus
IRB	Institutional review board
JVP	Jugular venous pressure
mPAP	Mean pulmonary artery pressure
PAH	Pulmonary arterial hypertension
PAWP	Pulmonary artery wedge pressure
PH	Pulmonary hypertension
PVR	Pulmonary vascular resistance
q.i.d.	Quarter in die
RAP	Right atrial pressure
RHC	Right heart catheterization
RHF	Right heart failure
RV	Right ventricle
RVSP	Right ventricular systolic pressure
sPAP	Systolic pulmonary artery pressure
SQ	Subcutaneous
t.i.d.	Ter in die
WHO	World Health Organization
WU	Wood units
6MWD	6 min walk distance
